# Supplementary material for: Effectiveness of a novel midwifery preceptor program on midwives’ competence and confidence in Sierra Leone: A prospective pilot study
Source: PLOS Glob Public Health. 2026 May 14;6(5):e0006294. doi: 10.1371/journal.pgph.0006294 (PMC13175337; doi:10.1371/journal.pgph.0006294)
Supplement: Attachment — Submitted filename: PLOS ONE_SL Midwifery effectiveness_response_v2.docx [file pgph.0006294.s001.docx]

January 31, 2026

Response to Reviewers

Dear Dr. Iqhrammaullah and reviewers,

Thank you for the opportunity to revise and resubmit our manuscript. Below we have addressed all reviewer comments point-by-point and greatly appreciate your feedback. We have detailed where in the manuscript we have made these changes and provided the manuscript in both tracked changes and a clean version for your review.

We believe these suggestions improved our manuscript. Thank you for your time and consideration.

Sincerely,

Brittney van de Water

**Response to Reviewers**

**Reviewer Comment 1.1:** Key words: Consider adding - Sierra Leone, Sub-Saharan Africa, low-resource country.

Response: Thank you. We have added these key words.

**Reviewer Comment 1.2**: Terminology: The authors used the word “significant improvement” throughout. Given the p-values when used, they may consider using “statistically significant improvement” when appropriate to distinguish from a subjective use of the term and to distinguish from “clinically significant”.

Response: Thank you. We have added “statistically significant improvement” when appropriate throughout the manuscript.

**Reviewer Comment 1.3**: Tables: Perhaps I missed this, but I could not find a sighting in the text for Table 6 and Table 7.

Response: Thank you. We have added the in-text citation for Table 6 (Student evaluations of preceptor) and Table 7 (Observed assessment of preceptor’s competence in precepting) on page 10 between Table 5 and Table 8.

**Reviewer Comment 1.4**: Discussion: Researcher direct observation: Lines 326 and 327 – “Direct observation of preceptors’ teaching competency did not show evidence of increased competency.” The authors explained that a low n value could explain this. Is it also possible that the preceptor program was not effective in changing preceptor teaching competency? If so, please add this as a possible explanation.

Response: Thank you for this suggestion. We have added to the discussion “Alternatively, the program may not have effectively improved preceptor competency.”

**Reviewer Comment 1.5**: Rater reliability: Did the same rater do all of the clinical observations? If so, please state this. If multiple raters were used, were there inter-rater reliability sessions and rater training?

Response: Thank you. No, the facilitators of the program (N=4) were Seed Global Health midwife educators and are visiting faculty at the schools of midwifery where the program was implemented. Rater trainings took place at the beginning of the program and throughout the program to ensure consistency in evaluations. Two authors (JM and BvdW) reviewed evaluations regularly and JM met bi-weekly with educators to discuss scoring. We have added “Across the two sites, there were four facilitators who rated participants. The four facilitators met regularly (at least bi-weekly) with the director of midwifery (JM) to discuss grading and ensure internal consistency in scoring assessments.” to the Tools/Procedures section.

**Reviewer Comment 2.1** [Title and abstract]
1. Given the very small sample size (N=20) and absence of a control group, the study is more appropriately classified as a pilot or feasibility study. The current title implies a level of evidentiary strength (“Effectiveness”) that is not supported by the design. Revise the title to explicitly reflect the pilot nature, “A pilot evaluation of a novel midwifery preceptor program…”

Response: Thank you. To stay in line with many *PLOS ONE* titles with the design after a colon or an em dash, we have changed the title to “Effectiveness of a novel midwifery preceptor program on midwives’ competence and confidence in Sierra Leone: a prospective pilot study”.

**Reviewer Comment 2.2** Objectives section of the abstract is unusually long and conceptually repetitive, blending background justification with aims. Rewrite the Objectives to be short, direct, and outcome-focused.

Response: Thank you. We have made the Objectives section one sentence now.

**Reviewer Comment 2.3.** The abstract does not sufficiently emphasize pilot nature of the study, small sample size, and lack of control group. Explicitly state in the abstract that this is a pilot/preliminary study. Avoid implying causal effectiveness; use cautious language such as “suggests improvement” rather than “demonstrates effectiveness.”

Response: Thank you. We now explicitly use the word “pilot” in the Background section of the abstract. We added “novel” in the Methods to briefly indicate, again, this was a pilot. We also state the sample size as n=10 per site for a total N=20. We also added “statistical” before “improvement”.

**Reviewer Comment 2.4** [Introduction] The section is lack of an explicit conceptual or theoretical framework guiding the intervention (e.g., competency-based education theory, adult learning theory, mentorship frameworks). Add a conceptual framework figure or narrative linking intervention components to expected outcomes.

Response: Thank you. To be more explicit about the low-dose, high-frequency conceptual model, we have added “This low-dose, high-frequency model has been shown to support maternal health interventions across Africa due to repeated sessions, simulation, team cohesion and its cost-effectiveness (13–16)” in the Study Design and Intervention section. If you prefer this in the Background, we are willing to move it up.

**Reviewer Comment 2.5** While prior needs assessments are cited, the novelty of the intervention relative to existing preceptor or mentorship programs is not clearly articulated.

Response: Thank you. We have added “This intervention differs from many existing training programs due to its low-dose, high-frequency model, low facilitator to preceptor ratio, program duration, and ongoing mentorship on a weekly basis throughout the program both through didactic and hands-on clinical experiential learning (18–20)” in Lines 179-183 of the Study Design and Intervention. Reference 18 details nine key components of this preceptor program and provides literature to back up the importance of each element in adult learning. Reference 20 detail the importance of Kolb’s experiential learning theory and how giving individuals time to experience, reflect, think, and act, individuals are better able to process all forms of learning, development, and change. Reference 19 provides a description of experiential learning that the author (van de Water) details with students in an unique study abroad experience that was more research- rather than class-based and afforded students significant time to experience, reflect, think, and act; similar to this preceptor program which blends didactic and clinical education.

**Reviewer Comment 2.6** The introduction does not sufficiently justify: (a) Why this specific intervention structure (low-dose, high-frequency) was chosen; (b) How it theoretically leads to improvements in competence and confidence. Authors should clearly articulate the theoretical mechanisms underlying competence and confidence improvement.

Response: Thank you. We believe responses to Comment 2.4 and 2.5 now justify part a. Additionally, we have now included our logic model (Figure 1) which is originally published in our protocol paper (Reference 13). This shows that with certain inputs (i.e, funding, facilitators, preceptor curriculum, appropriate tools and skills checklists, and guidelines) and planned activities (i.e., across midwifery institutions, clinical settings, preceptor course, a train-the trainer program, and certification of preceptors), there will be certain outputs (i.e., # of faculty trained, # of clinical sites with negotiated contracts, # of preceptors identified as eligible to participate), as well as outcomes (i.e., a change in clinical competence of preceptors, a change in teaching competence of preceptors, a change in hands-on time students receive with preceptors during clinical rotations, and a change in average objective structured clinical exam scores) and eventually impact (i.e., decreased maternal morbidity and mortality). Specifically, we added a brief sentence referencing this Figure at the end of the Study Design and Intervention which reads “The logical assumption underlying this program is that with sufficient inputs and planned activities, certain improved outputs and outcomes, as well as eventual long-term impact will occur (Figure 1) (13).”.

**Reviewer Comment 2.7** [Methods] No sample size calculation is reported. How were the 20 participants selected? Were inclusion/exclusion criteria applied? Was participation voluntary or assigned?

Response: Thank you. Correct – all participants in the Preceptor Program were included in analysis, so no sample size calculation was conducted. Under Population, we have added “Participants had to have a minimum of 2 years full-time clinical experience across the midwifery scope of practice within the last five years, have completed an accredited midwifery program and hold a current midwifery license, complete an application form and submit a letter of recommendation from a supervisor.”.

**Reviewer Comment 2.8** In the study design, frame the study clearly as exploratory/pilot.

Response: Thank you. We have added “The launch of the pilot-year of the program took place in February of 2023.” as the first sentence of the Study Design and Intervention.

**Reviewer Comment 2.9** The manuscript uses 25 different assessment tools, including written tests, OSCEs, self-assessments, and student evaluations. But, there is no clear description of validation, reliability testing, or prior use of these tools in similar settings. Cite prior validation, or internal consistency testing (such as Cronbach’s alpha), or at minimum, justification for tool development and adaptation.

Response: Thank you. OSCE, simulation and direct observation tools were developed using national protocols and evidence-based practice guidelines and were internally reviewed by midwifery faculty in Sierra Leone to ensure application to the local context. Teaching assessment tools were adapted from the American College of Nurse Midwives (ACNM, 2013). Self-assessment of Midwifery Competencies was developed from ICM Essential Competencies of Midwifery Practice (ICM, 2024); this has been added to the Tools/Procedures. In response to Comment 1.5 we added “Across the two sites, there were four facilitators who rated participants. The four facilitators met regularly (at least bi-weekly) with the director of midwifery (JM) to discuss grading and ensure internal consistency in scoring assessments.” to the Tools/Procedures section. Additionally, in the Limitations section we mention and cite “it is well established that OSCEs (in particular), are a valid and reliable form of assessment for examining competency after participating in a healthcare education program (50–53).”

**Reviewer Comment 2.10** Self-assessment measures are particularly vulnerable to social desirability and expectancy bias, especially in educational interventions. Explicitly discuss the limitations of self-reported outcomes.

Response: Thank you. Although self-assessment is particularly vulnerable to social desirability and expectancy bias, there was no other way to assess self-assessment of clinical precepting confidence and competence. Again, we had 25 different assessments, so these two self-assessments were to augment the 23 objective assessments. We also conducted qualitative interviews regarding preceptor and facilitator thoughts regarding the barriers and facilitators to the program in general, these ## self-assessments were used to triangulate quantitative findings (reported in a separate publication, which we cite, reference ##). Additionally, given how many tests the participants were given, asking them to assess themselves seemed appropriate and important, so as not to diminish their own self-reflection and evaluation, particularly regarding confidence. We have added to the limitations section “Two self-reported tools were used to help triangulate self-reported confidence. Although there are known limitations of self-report such as social desirability in students, self-report is also known to be the only way to report self-reported outcomes (53,54).”

**Reviewer Comment 2.11** Although the intervention is described in broad terms, key operational details are missing, including: (a) Exact curriculum content; (b) Facilitator training requirements; (c) Fidelity monitoring across sites.

Response: Thank you. We have added more regarding the curriculum content, facilitator training, and fidelity monitoring at the end of Study Design and Intervention. Specifically, we added “The curriculum consisted of 25 modules about ICM Essentials for Midwifery. Another 10 modules focused on adult learning theory and principles of precepting. Facilitators in year 1 of this program were all Seed Global Health Educators and had at least a bachelor’s or master’s degree in midwifery, went through a rigorous application and two-step interview process, had at least five years’ experience as a nurse-midwife, and were visiting faculty at the institutions in Sierra Leone where Seed had placed them. As mentioned previously, JM (Associate Director of Midwifery at Seed Global Health) had a bi-weekly meeting to discuss the preceptor program with the facilitators and spoke ad hoc with facilitators regularly and visited sites bi-annually with Seed country staff (MS, MB, IS) visiting at least quarterly.”.

**Reviewer Comment 2.12** [Results] Avoid overstating statistically significant findings. Interpret null findings more critically. Emphasize effect direction and feasibility, not effectiveness.
Response: Thank you. We have added additional interpretation of null findings in the Discussion; however, in line with *PLOS ONE* and most journal recommendations, we limit our interpretation of findings in the Results section. We appreciate the reviewer’s suggestion to focus on feasibility; however, we have chosen to maintain the focus on changes in confidence and competence for several reasons.

First, the feasibility of this intervention—including organizational readiness and implementation barriers—has been documented in our previous publications ([Ref 10], [Ref 12]). To include a detailed feasibility analysis here would result in redundant reporting of data already available in the literature.

Second, per the reviewer’s suggestion, we have added a brief mention of the 100% completion rate (20/20 midwives) as a proxy for participant-level feasibility. However, the primary aim of this specific analysis was to evaluate the impact of the pilot intervention on clinical mastery. Conflating implementation metrics (feasibility) with outcome metrics (effectiveness) would dilute the focus of this manuscript, which seeks to establish whether the program achieves its pedagogical goals (assessing confidence and competence).

Per the reviewer's suggestion, we have adjusted our language to emphasize effect direction (e.g., consistent improvements across all domains) to provide a more nuanced view of the results.

**Reviewer Comment 2.13** [Discussion] Causal language is used that is not supported by the design (limited sample/pilot). Authors must temper causal claims.

Response: Thank you. We have taken out the word “effective” and replaced the fourth paragraph with the sentence “This analysis demonstrates an association between increased pre- to post-assessment scores, indicating improved clinical competency and confidence of preceptors.”. We have added in language such as “association between” to differentiate between causal and noncausal relationships.

**Reviewer Comment 2.14** Theoretical explanations for observed changes (e.g., learning mechanisms, behavioral change pathways) are limited. Kindly discuss alternative explanations, including Hawthorne effect and maturation bias.

Response: Thank you. We have added the Hawthorne effect and maturation bias and references to the Limitations section. “Finally, alternative explanations for statistically significant findings could be biased due to phenomena such as the Hawthorne effect or maturation bias (54,55).”

**Reviewer Comment 2.15** The discussion does not sufficiently compare findings with similar preceptor or mentorship studies. Authors must expand comparison with global midwifery education literature.

Response: Thank you. We cite and compare findings with the Democratic Republic of Congo, Kenya, Ethiopia, Sierra Leone, a scoping review across the African continent, another across low- and middle-income countries, as well as Malawi and Zambia. Citations 30-49 are explicitly other nurse and midwifery preceptor-related programs. Given our extensive work in this area, this is quite an exhaustive list of references specific to nurse and midwifery education and training that is comparable to our findings. If there is anything in particular you would like us to compare, please let us know and we are happy to consider that interpretation.

**Reviewer Comment 2.16** Findings from 20 midwives in two schools cannot be generalized to: (a) Sierra Leone nationally; (b) Other low-resource settings. Clearly state that generalizability is very limited.

Response: Thank you. We state in the Limitations that N=20 is “a relatively small sample size, limiting the ability to conduct complex analyses and limiting generalizability”.

**Reviewer Comment 2.17** To strengthen the discussion on midwifery education as a core internal system strength, the authors are encouraged to cite: International Journal of Women’s Health. 2025;17:53–66. DOI: 10.2147/IJWH.S494922. This reference would help contextualize the importance of structured midwifery education interventions and reinforce the relevance of preceptor capacity-building.

Response: Thank you for this suggestion. We reviewed this reference and although very interesting that the significant role midwifery plays in providing maternal health services was a latent topic found in this analysis, this is also described by the ICM and the WHO globally. We feel our other 55 references are applicable to midwifery education and this preceptor program, particularly in sub-Saharan Africa. This article seems very specific to Indonesia and the Indonesian and Southeast Asian context as described in the use of the SWOT analysis. The conclusion explicitly states these ten latent topics should be focused upon “in Indonesia” and this paper seems out of context in relation to our intervention.

**Reviewer Comment 2.18** [Conclusion] The conclusion currently overreaches, implying effectiveness and policy applicability that are not supported by the study design. Revise conclusions to: (a) Emphasize feasibility and acceptability; (b) Acknowledge pilot limitations; (c) Recommend for larger, controlled studies. If conclusions remain overstated, this alone would justify rejection.

Response: Thank you. We have adjusted and add to the Conclusion to now read “While this pilot study was limited in scale, we observed a consistent positive increase across pre- to post-intervention assessments. These preliminary findings suggest that this low-dose, high-frequency preceptor program is a promising model for strengthening midwifery education in low resource settings. To build on this proof-of-concept in two sites, future large-scale, multi-site studies are essential to establish the generalizability of these outcomes and to evaluate the long-term impact of these clinical competencies on maternal health outcomes.”
